# TNF-α mRNA is negatively regulated by microRNA-181a-5p in maturation of dendritic cells induced by high mobility group box-1 protein

**DOI:** 10.1038/s41598-017-12492-3

**Published:** 2017-09-25

**Authors:** Jing Zhu, Fu-Li Wang, Hai-Bin Wang, Ning Dong, Xiao-Mei Zhu, Yao Wu, Yong-Tao Wang, Yong-Ming Yao

**Affiliations:** 1 Trauma Research Center, First Hospital Affiliated to the Chinese PLA General Hospital, Beijing, 100048 P.R. China; 2 Department of Clinical Laboratory, First Hospital Affiliated to the Chinese PLA General Hospital, Beijing, 100048 P.R. China; 3 Department of Emergency Medicine, Tianjin Medical University General Hospital, Tianjin, 300052 P.R. China; 4 State Key Laboratory of Kidney Disease, the Chinese PLA General Hospital, Beijing, 100853 P.R. China

## Abstract

Dendritic cell (DC) can be stimulated by both exogenous pathogen-associated molecular patterns (PAMPs) such as lipopolysaccharide (LPS) and endogenous damage-associated molecular patterns (DAMPs) such as high mobility group box-1 protein (HMGB1). MicroRNAs (miRNAs) act as post-transcriptional fine tuners of mRNA. Studies have focused mostly on the potential role of miRNAs in DCs maturation triggered by PAMPs, especially LPS, however, little is known about the regulatory mechanism underlying the effects of miRNAs in DC maturation mediated by DAMPs, including HMGB1. Here, we first profiled a miRNA microarray of DCs stimulated by HMGB1 and determined that the up-regulated miRNA miR-181a-5p may act as a regulatory miRNA in these cells. Computational algorithms predicted TNF-α 3′UTR to be targeted by miR-181a-5p, which was confirmed by the experiments involving luciferase reporters. In addition, we found that TNF-α mRNA was down-regulated by miR-181a-5p mimic, and significantly up-regulated by miR-181a-5p inhibitor. Taken together, we identified miR-181a-5p a negative regulator in HMGB1-induced immune responses by targeting TNF-α mRNA in DCs. Moreover, we suggested that miR-181a-5p may play a role in regulating DC responses to HMGB1 and serve as evidence indicating that novel therapies targeting miRNAs may be useful for treating immune dysfunction in the setting of sepsis.

## Introduction

Sepsis, a major complication of burns and trauma, remains a common cause of death^1^. Recent studies have suggested that the occurrence and development of severe sepsis is closely related to host immune dysfunction^2,3^. Dendritic cells (DCs) play important roles in the immune response and are potent antigen-presenting cells (APCs) that primarily act as a bridge between the innate and adaptive immune systems^4,5^.

Classically, danger signals have been defined as exogenous, pathogen-associated molecular patterns (PAMPs), such as the components of pathogens [e.g., lipopolysaccharide (LPS) and peptidoglycan]^6^, and such signals can induce inflammatory and immune response. It has been documented that endogenous molecules released from injured cells, so-called damage-associated molecular patterns (DAMPs), or alarmins, such as high mobility group box-1 protein (HMGB1), which was originally described as a DNA-binding protein in the nucleus^7^, can also trigger host immune dysfunction^8^. In addition to being released passively, HMGB1 can also be secreted actively by macrophages, natural killer (NK) cells, and DCs to act as a late mediator of sepsis^9,10^. The results of a series of previous scientific observational studies focusing on HMGB1^11–14^ have indicated that extracellular HMGB1 plays a very important role in mediating DC maturation and differentiation, Th1 polarization and immune response induction *in vitro*. Moreover, the results of previous animal experiments and clinical investigation studies conducted in our lab suggested that HMGB1 has dual regulatory effects on the immune function of T lymphocytes, DCs and macrophages both *in vivo* and *in vitro* ^15,16^. Our findings clearly demonstrated that excessive release of HMGB1 contributes to the development of immune dysfunction in sepsis. However, the precise molecular mechanisms underlying the regulatory effect of HMGB1 on cell-mediated immunity remain to be elucidated.

MicroRNAs (miRNAs) are a class of small, endogenous noncoding RNA molecules that act as post-transcriptional fine tuners to mediate inhibition^17,18^ or activation^19–21^ of mRNA translation to regulate protein expression. miRNAs are involved in almost every cellular process, including proliferation, differentiation, and apoptosis. Moreover, studies have shown that miRNAs profiles changes significantly during the immune response^22–24^. The importance of miRNAs in the host immune system has been recognized by increasing numbers of researchers; however, the role of miRNAs in the regulation of HMGB-mediated immune dysfunction in sepsis remains unknown.

Here, we first validated the effects of HMGB1 on the maturation and activation of splenic DCs from mice. We then profiled a miRNA microarray of DCs stimulated by extracellular HMGB1 and identified fourteen miRNAs that were differentially expressed between DCs incubated with HMGB1 and DCs incubated without HMGB1. Based on a literature report and computational predictions, we determined the up-regulated miRNA miR-181a-5p may act as a regulatory miRNA in DCs. Computational algorithms predicted that the 3′UTR of tumor necrosis factor (TNF)-α was targeted by miR-181a-5p. This prediction was confirmed by experiments involving luciferase reporters. In addition, we performed experiments in which miR-181a-5p was up- and down-regulated by mimics and inhibitors. These experiments confirmed that miR-181a-5p regulates TNF-α mRNA expression.

## Results

### HMGB1 induced DC maturation and activation

#### HMGB1 induced phenotypic maturation and cytokine expression in DCs

In our previous studies^15^, we found that the HMGB1 may regulate DC maturation and activation of DCs in rats. Herein, we attempted to confirm that HMGB1 has immune regulatory effects on DCs from mice and to determine the appropriate extracellular HMGB1 concentration with which to stimulate DCs *in vitro*.

**Figure 1:**
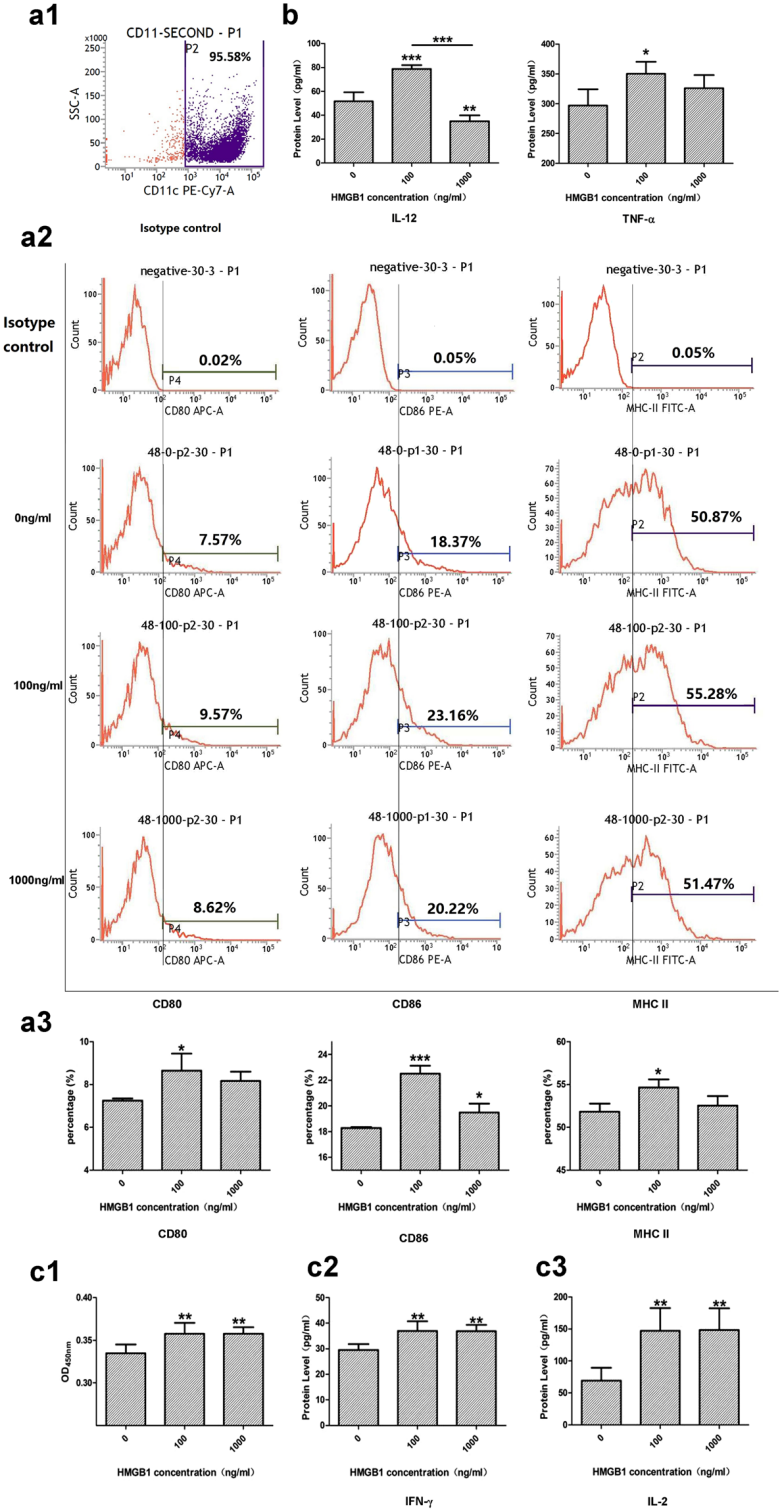
HMGB1 induced DC maturation and activation. Immature splenic DCs were cultured with HMGB1 at the indicated doses (100 and 1000 ng/ml) or without HMGB1 (0 ng/ml, as control) for 48 hours. (**a1**) Flow cytometry gating strategy for CD11c^+^DC cells with the purity of 95.58% after twice MS colume separations. (**a2**) Data shown are representative histograms of CD80, CD86 and MHC II, using FACS with APC-, PE- or FITC-conjugated antibodies, respectively. The gate was set according to the data of unstained negative group (Isotype control). (**a3**) The percentage of DCs expressing CD80, CD86, and MHC II was significant elevated after 100 or 1000 ng/ml HMGB1 stimulating and peaked in the concentration of 100 ng/ml. Results of 3 independent experiments were shown as the mean ± SEM. (**b**) The levels of IL-12 and TNF-α secreted by DCs stimulated with HMGB1 were measured via ELISA. Results of 3 independent experiments were shown as the mean ± SEM. T cells were incubated with ConA (5 μg/ml) for 24 hours, and co-cultured with stimulated DCs at a ratio of 1:100. (**c1**) A CCK-8 cell proliferation assay was used to assess T-cell proliferative activity after co-culture for 68 hours. (**c2**) Meanwhile, using ELISAs, the levels of IFN-γ in culture medium were determined to evaluate Th1 and Th2 polarization. (**c3**) The levels of IL-2 in cell culture supernatants were also measured. Results of 3 independent experiments were shown as the mean ± SEM. *P < 0.05, **P < 0.01, ***P < 0.001 vs. 0 ng/ml group.

As shown in Fig. 1a1–a3, stimulating DCs with 100 or 1000 ng/ml HMGB1 for 48 hours up-regulated the percentage of DCs which express CD80, CD86 and MHC-II. The difference in the percentage of DCs expressing the indicated molecules between the HMGB1-treated and control groups was statistically significant (*P* < 0.05 or *P* < 0.001) and the percentage of DCs expressing all three molecules were respectively dual-regulated (up-regulated when 100 ng/ml HMGB1 treated and dropped when HMGB1 was 1000 ng/ml).

To assess the effects of HMGB1 on the expression levels of the cytokines reflecting DC maturation, we measured interleukin (IL)-12 and TNF-α protein levels in the supernatants of mice splenic DCs treated with or without HMGB1. As shown in Fig. 1b, stimulating DCs with HMGB1 (100 or 1000 ng/ml) for 48 hours resulted in significant increase in TNF-α production in DCs in the indicated group compared with the control group (*P* < 0.05). However in the case of IL-12, only treatment with 100 ng/ml HMGB1 resulted in increases in its production (*P* < 0.001). Treatment with 1000 ng/ml HMGB1 resulted in decreased IL-12 production(*P* < 0.01). In addition, we noted that treating DCs with 100 ng/ml HMGB1 for 48 hours resulted in greater increases in IL-12 and TNF-α production than treating DCs with 1000 ng/ml HMGB1, finding that paralleled those pertaining to the experiments in which the percentage of DCs expressing the phenotypic molecules on the surface of DCs were assessed, showing that HMGB1 possessed a dual influence on the phenotypic maturation and cytokine expression in DCs.

#### DCs stimulated by HMGB1 promoted T cell proliferation, differentiation and cytokine expression

We analyzed HMGB1-treated DCs to assess their capacity to stimulate Concanavalin A (ConA) (T-cell mitogen)-mediated T cell proliferation and differentiation, a parameter that serves as an index of DC functional maturation (Fig. 1c). CCK-8 cell proliferation assay showed that DCs treated with HMGB1 (100 and 1000 ng/ml) for 48 hours, induced significant increases in ConA-mediated T cells proliferations compared with control cells (*P* < 0.01) when the two cell types were cultured together at a 1:100 DC:T ratio over 68 hours, as shown in Fig. 1c1. We also measured interferon (IFN)-γ levels in the culture medium by ELISA to evaluate Th1 and Th2 polarization. IL-2 acts upon itself in an autocrine manner and is a potent T-cell growth factor. We assessed IL-2 expression in T cells co-cultured with HMGB1 (100 and 1000 ng/ml for 48 hours)-treated DCs. We found that IFN-γ and IL-2 expression levels in splenic T cells co-cultured with HMGB1-pretreated DCs at a DC:T ratio of 1:100 for 68 hours were significantly enhanced compared to with those in control DCs (without HMGB1, 0 ng/ml-pretreated DCs) (*P* < 0.01), as shown in Fig. 1c2 and 1c3. In addition, the results were in agreement with previous observations showing that HMGB1 possessed a dual influence on DCs stimulating T cells. Thus, we selected 100 ng/ml as the appropriate concentration for HMGB1 to induce the maturation of DCs.

### Profiling and identification of miR-181a-5p as a potential regulatory miRNA in DCs

We analyzed the expression profile of miRNAs in mice splenic DCs stimulated by HMGB1. We used an Agilent Mouse miRNA Microarray, release 21.0, platform containings 1881 mouse miRNAs for this experiment. We normalized the raw data and determined that fourteen miRNAs were differentially expressed between DCs incubated with and DCs incubated without HMGB1. The corresponding absolute fold change threshold and *P* value were ≥1 and ≤0.05, respectively. As shown in Table 1, miR-181a-5p expression level in the HMGB1-treated group was 1.0242-fold higher than that of the control group.

**Table 1:** miRNAs differentially expressed in response to HMGB1 treatment according to microarray data.

Up-regulated			Down-regulated		
miRNA name	Fold change	P value	miRNA name	Fold change	P value
miR-3085-5p	1.2218	0.0092	miR-6395	0.7485	0.0205
miR-351-3p	1.1703	0.0327	miR-7020-3p	0.8684	0.0132
miR-2861	1.1486	0.0162	miR-6984-3p	0.9002	0.0408
miR-8102	1.0625	0.0244	miR-7233-5p	0.9040	0.0177
miR-6954-5p	1.0577	0.0281	miR-5620-5p	0.9082	0.0336
miR-7686-5p	1.0346	0.0351	miR-30c-1-3p	0.9219	0.0143
miR-181a-5p	1.0242	0.0323	miRNABrightCorner30	0.9520	0.0136

Fourteen differentially expressed miRNAs were differentially expressed between DCs incubated with HMGB1 and DCs incubated without HMGB1. The corresponding absolute fold change threshold and *P* value were ≥1 and ≤0.05, respectively. Of the miRNAs, seven were significantly up-regulated and seven were significantly down-regulated.

After reviewing the literature, we recognized that miR-181a-5p plays an important role in the development of sepsis^25^ and the modulation of DCs^26^. Our first experiments clearly demonstrated that excessive HMGB1 release might contribute to DC maturation and activation; thus, in our subsequent experiments, we focused on miR-181a-5p and sought to predict its target genes using miRANDA. The indicated program identified 487 putative target genes of miR-181a-5p. One of the target genes was TNF-α.

**Figure 2:**
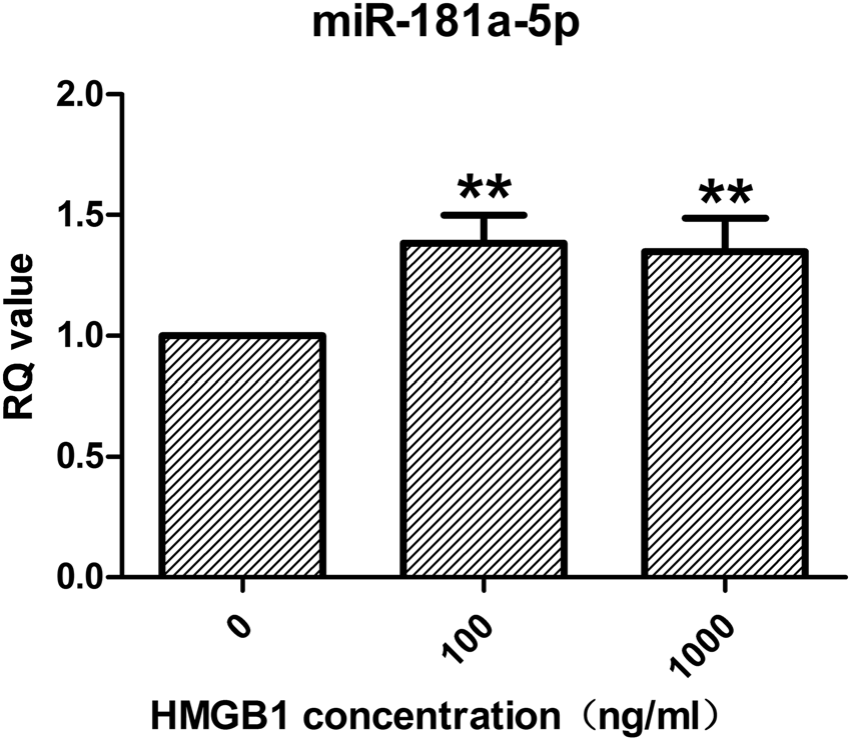
Validation of the microarray expression patterns of miR-181a-5p in DCs stimulated by HMGB1. Immature DCs were cultured with the indicated doses of HMGB1 (100 and 1000 ng/ml) or without HMGB1 (0 ng/ml, control). The up-regulation of miR-181a-5p in DCs stimulated by HMGB1 (100 and 1000 ng/ml) was significant compared with the DCs cultured without HMGB1 (0 ng/ml, as control). Results of 3 independent experiments were shown as the mean ± SEM. **P < 0.01 vs. 0 ng/ml group.

We subsequently focused on validating the microarray expression patterns of miR-181a-5p by quantitative real-time PCR (RT-PCR), which showed that the expression level of miR-181a-5p in DCs stimulated by HMGB1 (100 ng/ml) (Fig. 2) was 1.38-fold higher than that in control group, and that, the expression pattern of miR-181a-5p noted in our experiments was consistent with that noted in the miRNA microarray data.

### TNF-α was a direct target of miR-181a-5p

**Figure 3:**
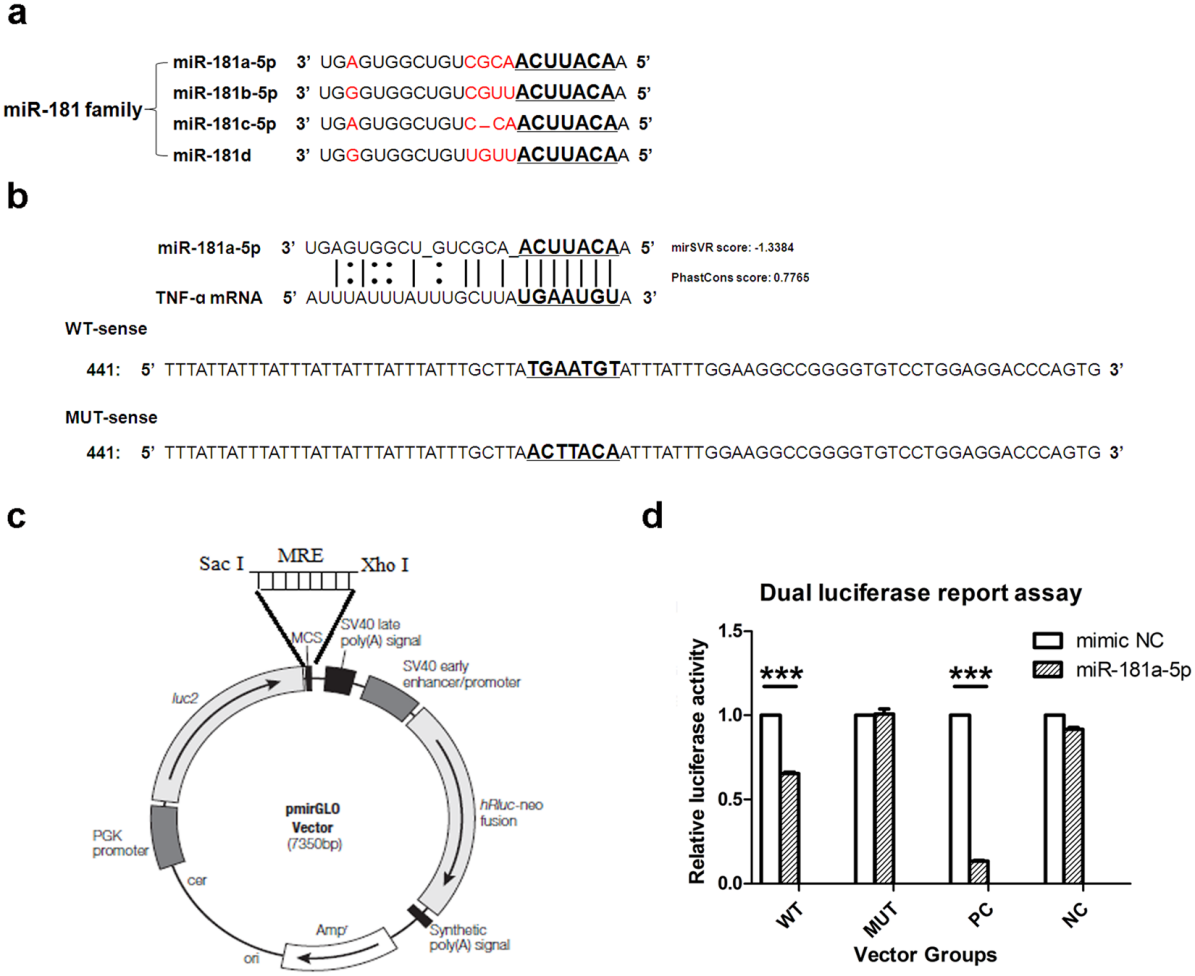
TNF-α mRNA was a direct target of miR-181a-5p. (**a**) The mature sequences of miR-181 family members that arise from the 5′ arm of the precursors contain similar seed sequences (as shown in bold with underlining). Red color indicates the different nucleotides of each miR-181 family member. (**b**) The high scores of the conserved TNF-α 3′UTR as a computationally predicted target gene of miR-181a-5p. The mirSVR Score and the PhastCons score reflect the effect of the miRNA acting on mRNA and the conservatism of the predicted binding sites in the target mRNA, respectively. The 80 bp sequence of the TNF-α-3′UTR containing the predicted miR-181a-5p binding sites (the WT sense) and its mutant (the MUT sense) were synthesized for cloning into a pmirGLO Vector. (**c**) Dual-luciferase assay (based on the illustration of the pmirGLO vector multiple cloning site provided in the manufacturer’s instructions). Schematic of pmirGLO Dual-Luciferase miRNA Target Expression Vector (Promega) used to construct TNF-α-3′UTR- based miRNA reporter plasmids. The dual-luciferase vector contains both modified firefly (hluc^+^) and Renilla (hRluc) luciferase genes. The desired miRNA recognition elements (MRE) were cloned into SacI-XhoI sites in the multiple cloning site (MCS) located in the 3′UTR of the hluc gene. (**d**) Luciferase activity in 293 T cells transfected with four types of vectors including TNF-α-pmirGLO (WT) or mutant of TNF-α-pmirGLO (MUT), two duplicates of the miR-181a-5p inhibitor-pmirGLO (PC) and empty pmirGLO (NC) plus mimic NC and mmu-miR-181a-5p. Results of 3 independent experiments were shown as the mean ± SEM. ***P < 0.001 vs. mimic NC group.

The miR-181 family consists of the following four members: miR-181a, miR-181b, miR-181c and miR-181d. The two mature miRNAs generated from the 5′ or 3′ arms of the miRNA precursor are denoted with a -5p or -3p suffix, respectively. miR-181a-5p is a mature miRNA generated from the 5′ arms of the miR-181a precursor and contains the seed sequence shown in Fig. 3a. Based on a literature report^25^ and computer-determined scores (mirSVR Score: −1.3384 and the PhastCons score: 0.7765) indicating that the conserved TNF-α 3′UTR may be a target gene of miR-181a-5p (Fig. 3b), we hypothesized that miR-181a-5p might target the TNF-α 3′UTR.

To test this hypothesis, we used a pmirGLO Dual-Luciferase miRNA Target Expression Vector (Promega) containing modified versions of both Renilla reniformis (sea pansy) luciferase (hRluc) and Photinus pyalis (firefly) luciferase (hluc) (Fig. 3c).

The transfection efficiency was more than 70% (see Supplementary Fig. S1A). As shown in Fig. 3d, the WT cotransfectants exposed to miR-181a-5p displayed a significantly lower luciferase activity level than those exposed to the mimic negative control (mimic NC). Specifically, the luciferase activity level in the former group was 65.3% of that in the latter group. However, miR-181a-5p did not significantly modify the luciferase activity of the MUT cotransfectants. These data indicated that miR-181a-5p can bind the 3′UTR of TNF-α mRNA to negatively regulate its expression.

### miR-181a-5p inhibiton and overexpression regulated TNF-α mRNA in DCs

**Figure 4:**
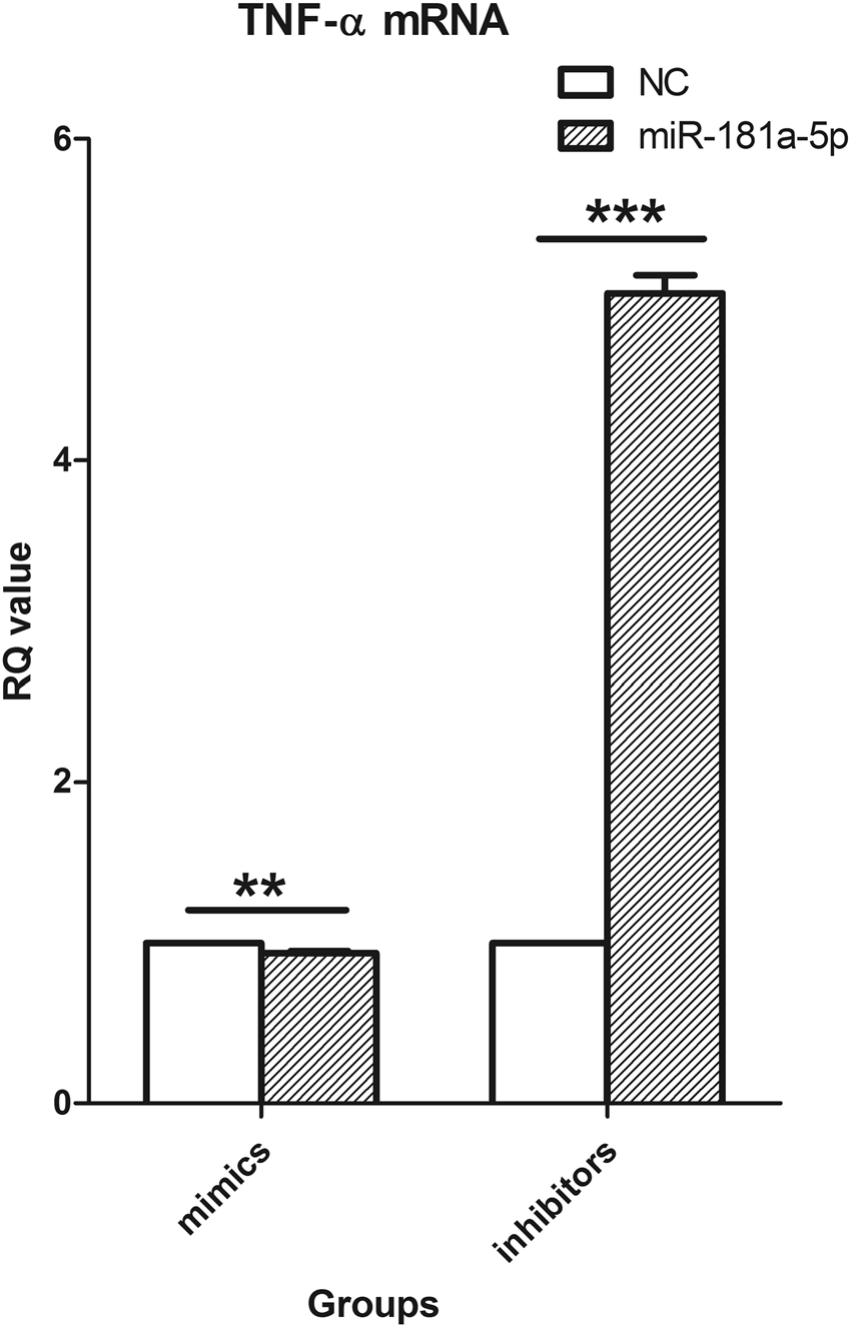
TNF-α mRNA was regulated by miR-181a-5p in DCs. Mice splenic DCs were transfected with miR-181a-5p mimic, mimic negative control (mimic NC), miR-181a-5p inhibitor and inhibitor negative control (inhibitor NC). After 48 hours, these cells were collected and TNF-α mRNA was measured using RT-PCR and normalized to the expression of β-actin mRNA in each sample. Results of 3 independent experiments were shown as the mean ± SEM. **P < 0.01 vs. mimic NC group. ***P < 0.001 vs. inhibitor NC group.

The transfection efficiency was more than 70% (see Supplementary Fig. S1B1, S1B2). DCs were transfected with miR-181a-5p mimic, mimic negative control (mimic NC), miR-181a-5p inhibitor and inhibitor negative control (inhibitor NC) and then collected approximately 48 hours later. After the total RNA was abstracted and reverse transcribed into cDNA, the expression of TNF-α mRNA, a target mRNA of miR-181a-5p, was determined by RT-PCR. The results showed as Fig. 4, revealed that the miR-181a-5p mimic suppressed TNF-α mRNA expression (*P* < 0.01) and the miR-181a-5p inhibitor significantly up-regulated TNF-α mRNA expression in DCs (*P* < 0.001).

## Discussion

Previous studies have shown that the occurrence and development of severe sepsis is closely related to host immune dysfunction. The initial hyperinflammatory state soon develops into an immunosuppressive state^1,27^. As professional APCs and coordinators of the innate and adaptive immune responses, DCs are very important immune cells. Completely activated DCs acquire a mature phenotype, characterized by up-regulation of certain surface molecules that are essential for antigen presentation, a change in chemokine responsiveness, and production of a variety of inflammatory cytokines as well as chemokines^4^.

DCs can be stimulated by danger signals including exogenous and endogenous signals. Previous reports have mostly examined the role of miRNAs in DC maturation triggered by PAMPs, especially LPS^28–32^. In contrast, we are the first to demonstrate a mechanism by which miRNAs modulations the maturation of DCs triggered by DAMPs, specifically HMGB1. When sepsis occurs, various types of cells are damaged, and then HMGB1 is then passively released from the nucleus of the necrotic cells^33^. HMGB1 is also actively secreted by macrophages, NK cells, and DCs; acts as a late mediator of sepsis^9^; and plays a very important role in immune dysfunction. In the present study, we verified the effects of HMGB1 stimulation in DCs from mice. Specifically, we found that HMGB1 stimulation in DCs results in up-regulation of the percentage of DCs expressing the indicated phenotypic molecules CD80, CD86, and MHC-II on the surface of DCs; simultaneous increase in the expression and release of IL-12 and TNF-α; and increases in T cell proliferation and differentiation, findings similar to those reported in our previous study, which involved rats^15^. HMGB1 was confirmed to be a potential immune-stimulatory signal that induces DC maturation and activation, a result that corroborated those of other reports^11–14^. However, the stimulatory effects of HMGB1 on DC maturation did not follow a linear pattern. Our previous studies about time- and dose-course showed that the highest levels of phenotypic and functional activation of DCs were observed at 48 hours when rats DCs were treated with 1000 ng/ml HMGB1^15^ and at 48 hours when mice DCs were treated with 100 ng/ml HMGB1 (see Supplementary Fig. S2). In addition, Isabella *et al*.^34^ performed time-course RT-PCR experiments and indicated that miR155 accumulation was increased progressively, reaching maximal levels at 48 hours of stimulation human Mo-DCs with LPS. All that indicated the time of 48 hours is potentially important both in the phenotypic and functional activation of DCs and in the expression of mature miRNAs. Based on a preliminary experiment, here, we treated the mouse splenic DCs 48 hours with 100 ng/ml and 1000 ng/ml HMGB1 respectively. In our results, a higher level of DCs maturation and activation in mice was noted under 100 ng/ml stimulation, of which the concentration is covered by the range equivalent to what is found *in vivo* in sepsis induced by LPS in mice^9^. The dual regulatory effects supported the formerly stated nonlinear hypothesis. Nonetheless, the precise mechanisms underlying the molecular regulatory role of HMGB1 in cell-mediated immunity remains to be elucidated.

We profiled a miRNA microarray of DCs simulated by 100 ng/ml extracellular HMGB1 for 48 hours and found that fourteen miRNAs were differentially expressed between DCs incubated with HMGB1 and DCs incubated without HMGB1. Based on a literature report^25^ and a computational prediction, we identified the up-regulated miRNA miR-181a-5p as a potential regulatory miRNA in DCs. Additionally, we confirmed that miR-181a-5p expression levels are up-regulated in DCs stimulated by HMGB1 by RT-PCR. miR-181a-5p is a member of the miR-181 family, which consists of four subtypes: miR-181a, miR-181b, miR-181c, and miR-181d. miR-181a-5p is a mature sequence generated from the 5′ arm of the precursor of miR-181a. Although -5p miR-181 family members have the same seed sequence, they have distinct gene targets^35^. Recently, miR-181a was highlighted in endothelial cells^36^, vascular smooth muscle cells^37^ and leukocytes^35^. Especially in leukocytes, miR-181a plays a very important regulatory role in B-cell differentiation^38^; T cell development^39^, maturation^40^ and sensitivity^39,41^; NKT cell development^42,43^; and DC function^26^. Nevertheless, little is known about the possible role of miR-181a-5p in immune regulation of DCs.

Wu *et al*.^26^ reported that miR-181a acts as a negative regulator in ox-LDL-induced inflammation via targeting of the 3′UTR of c-Fos in DCs. In the current experiments, we identified miR-181a-5p as a negative regulator in the HMGB1-induced immune response via targeting of TNF-α mRNA in DCs. Thus, both studies showed that miR-181a acts as a negative regulator in inflammation. In addition to DCs, a similar conclusion can be drawn in human monocytes, THP-1 cells and A549 cells in Dan *et al*.’s study^25^. They have verified that miR-181s family members, including miR-181a were up-regulated and play as negative regulators in LPS or ouabain-mediated inflammation via targeting of TNF-α mRNA. Taken together, these findings indicate that miR-181a up-regulation may act to protect the host against inflammation, thereby contributing to a shift of the initial hyperinflammatory state to the later immunosuppressive state; however, the mechanism underlying this phenomenon must be elucidated in further works. In addition, our data showed that in inflammation (including TNF-α up-regulation), miR-181a expression levels increased in DCs induced by stimuli, findings consistent with those of the report by Wu *et al*.^26^. However, our finding that TNF-α protein expression levels were also up-regulated in inflammation is inconsistent with our other data, which predicted that miR-181a-5p up-regulation results in TNF-α mRNA down-regulation. We propose that TNF-α protein expression is regulated by numerous factors and miR-181a-5p acts as a “fine-tuner” of its expression rather than as a major determinant of its expression. Furthermore, our previous *in vitro* experiments^15^ have shown that the TNF-α protein expression increased in rats DCs induced by HMGB1 for 24 h, 48 h and 72 h and peaked at 48 h. The TNF-α protein expression at 72 h decreased markedly compared to that at 48 h. *In vivo*, as an “early” mediators, TNF-α is released within minutes after LPS exposure and returned to basal levels about 4 h later. All that indicated, as sepsis developing, TNF-α levels first elevate and then decreased rapidly. What is the underlining regulatory mechanism? Maybe the negative regulator, miR-181a plays a very important role in this process.

Recently, Meike *et al*.^44^ reported that miR-181a also influenced HMGB1 expression in acute leukemias. When sepsis occurs, HMGB1, which regulates miR-181a expression, may also be regulated by miR-181a, and the two molecules may form a complex network to fine-tune cellular function and thus maintain immune homeostasis.

Although we are not the first to observe the TNF-α 3′UTR being targeted by miR-181, there are still important values of our findings. In our present study, we first profiled the miRNAs expression and identified up-regulated miR-181a-5p targeting the TNF-α mRNA in mice primary splenic DCs induced by HMGB1, compared the Dan *et al*.’s study^25^ showing the results in human cells. We two studies are important and meaningful for a better understanding of the roles of miR-181s family members both in human and in mice. Furthermore, our findings might lay a theoretic foundation for subsequent work to illustrate the underlining mechanism of immune dysfunction of sepsis. In summary, we determined that miR-181a-5p acts as a negative regulator in HMGB1-induced immune response by targeting of TNF-α mRNA in DCs. Our findings indicated that miR-181a-5p may play a role in regulating DC response to HMGB1 and serve as evidence supporting the idea that targeting miRNAs may represent a novel strategy for treating of immune dysfunction in the setting of sepsis.

## Materials and Methods

### Reagents

Mouse spleen lymphocyte separation medium and PBS were purchased from HaoYang Technology Co. Ltd., Tianjin, China. RPMI 1640 was purchased from Hong Wei Biotech Inc., Shijiazhuang, China. Fetal calf serum (FCS) was purchased from Gibco Co., CA, USA. The complete medium used throughout the experiments was RPMI 1640, supplemented with 10% FCS. Opti-MEM medium was purchased from Gibco Co. Recombinant HMGB1 was purchased from R&D Systems, Minneapolis, USA. CD11c^+^ (N418) MicroBeads were purchased from Miltenyi Biotec GmbH, Bergisch Gladbach, Germany. ConA was purchased from Sigma, St. Louis, MO. The antibodies used for flow cytometry analysis, including FITC anti-mouse MHC-II, PE anti-mouse CD86 antibody, and APC anti-mouse CD80 antibody were purchased from Miltenyi Biotec GmbH. ELISA kits for IL-12, TNF-α, IL-2, and IFN-γ were purchased from ExCell Biotech (Taicang) Co., Ltd., Shanghai, China. CCK-8 cell proliferation assay was purchased from Sigma, St. Louis, MO. The TRIzol reagent was purchased from Invitrogen, USA. The reverse transcription system were purchased from Promega, Madison, WI. miRNA first strand cDNA synthesis (Poly A Tailing) kit was purchased from Sangon Biotech, Shanghai, China. SYBR Green PCR Master MIX was purchased from KAPA Biosystems, Boston, MA. Lipofectamine 2000 was purchased from GenePharma Biotech Co., Ltd, China.

### Mice and DCs

C57BL/6J mice (aged 6–8 weeks) were provided by the Laboratory Animal Center, Chinese Academy of Medical Sciences, Beijing, China. The animals were housed in separate cages in a temperature-controlled room under a 12-hour light/dark cycle and were allowed to acclimatize to their environment for at least 7 days before being used in the experiments. All the animals had free access to water but were fasted overnight before the experiments. All experimental ental procedures were in accordance with the Guide for the Care and Use of Laboratory Animals published by National Institutes of Health, and with the approval of the Scientific Investigation Board of the Chinese PLA General Hospital, Beijing, China.

Using aseptic technique, we harvested the spleens of normal mice and washed them with ice-cold PBS for twice. Mononuclear cells were subsequently isolated from spleen preparations with mouse spleen lymphocyte separation medium, as previously described. Splenic DCs were isolated from the indicated mononuclear cells by positive selection using CD11c^+^ MicroBeads (10 μl/10^7^ total cells) and a Mini-MACSTM Separator with a positive selection MS column, according the manufacturer’s instructions. The selected DCs were subsequently pelletized by centrifugation (300 × g, 10 minutes), after which the supernatant was discarded, and the cell pellet was resuspended in the desired volume of RPMI 1640 FCS (10%) medium before being cultured in a humidified incubator overnight at 37 °C with 5% CO_2_ to recover. The purity of splenic CD11c^+^ DCs used for the experimentwas greater than 95% as determined by flow cytometry (see Supplementary Fig. S3). Trypan blue staining was used to count the alive cells and the viability of splenic DCs was greater than 95%.

### Isolation of T lymphocytes

CD4^+^ T cells were stained with biotin-antibody cocktail (10 μl/10^7^ total cells) and incubated for 10 minutes at 4 °C. They were then magnetically labeled with anti-biotin microbeads (20 μl/10^7^ total cells) and then, incubated for 15 minutes at 4 °C, after which they were harvested through a negative selection MS column (purity of purified CD4^+^ T cells >90%).

### Cell culture and stimulation

Polymyxin B (10 µg/ml) was added to the RPMI 1640 to neutralize the activity of endotoxin. DCs were resuspended with pretreated RPMI 1640 containing 10% FCS at a density of 2 × 10^6^ cell/ml, and then plated onto cell culture plates and cultured in a humidified incubator at 37 °C with 5% CO_2_. Recombinant HMGB1 was added to the medium at the indicated concentrations (0, 100, 1000 ng/ml), and the cells were cultured for 48 hours. The stimulation conditions were determined based on the results of our previous experiments^15^ and other published studies^45^. After being cultured and stimulated as indicated above, the cells were collected for flow cytometric analysis, RT-PCR or co-culture with T cells, and the culture supernatant was collected for cytokine expression analysis via ELISA.

### Flow cytometric analysis

DCs (1 × 10^5^) were reacted in 100 µl of PBS containing 5% FCS, 0.1% sodium azide (staining buffer) as well as APC-conjugated IgG specific for CD80, PE-conjugated IgG specific for CD86 or FITC-conjugated IgG specific for MHC-II, for 15 minutes at 4 °C. The cells were then washed twice with PBS (pH 7.2–7.4) containing 5% FCS and 0.4% paraformaldehyde, before being examined by flow cytometry using a FACScan flow cytometer (BD Biosciences, Mountain View, CA).

### T-Cell proliferation assay

The proliferation rates of T cells that had been co-cultured with DCs stimulated by different concentrations (0, 100, 1000 ng/ml) of HMGB1 for 48 hours were assessed by CCK-8 cell proliferation assay. The T cells were plated in 96-well plates at a density of 2 × 10^5^ cells/well, incubated with the T-cell mitogen Con A (5 µg/ml) for 24 hours, mixed with or without the indicated DCs at a DC:T ratio of 1:100 or without DCs, and then cultured for 68 hours. CCK-8 solution (10 µl/well) was then added to the mixture, and the incubation was continued for 4 hours. The optical density was measured at a wavelength of 450 nm using a microplate reader (Biotek, Winooski, USA).

### Cytokine measurements by ELISA

Cytokines levels, including IL-12, TNF-α, IFN-γ and IL-2 levels, in the cell culture supernatants were determined by ELISA, in strict accordance with the protocols provided by the manufacturer. The color reaction was terminated via the addition of 100 µl of ortho-phosphoric acid. The plates were read at a wavelength of 450 nm using a microplate reader (Biotek, Winooski, USA).

### SYBR green real-time RT-PCR

After the cells were treated as indicated, total RNA was extracted by cells using the single-step technique of acid guanidinium thiocyanate-phenol-chloroform extraction according to the manufacturer’s instructions (Invitrogen). The concentration of purified total RNA was determined spectrophotometrically at a wavelength of 260 nm. After the removal of potentially contaminating DNA with DNase I, 1 µg of total RNA from each sample was reverse transcribed with oligo dT and Superscript II to generate first strand cDNA. Mouse TNF-α mRNA expression levels were quantified in duplicate with SYBR green two-step, real time RT-PCR. The expression levels of β-actin, which served as an internal control, were determined under the same conditions using the appropriate primers. The PCR reaction mixture was prepared with SYBR Green PCR Master Mix using the primers shown in Table 2. The following thermal cycling conditions were used for the experiment, which was performed on a Sequence Detection System: 3 minutes at 95 °C, followed by 40 cycles of 95 °C for 3 seconds and 60 °C for 30 seconds. TNF-α expression levels were normalized to β-actin mRNA expression levels..

**Table 2:** Primer sequences used for polymerase chain reaction (PCR).

Target gene	Oligonucleotide primer (5′-3′)	
TNF-α	sense	CCACGCTCTTCTGTCTACTGAACT
	antisense	GGGTCTGGGCCATAGAACTG
β-actin	sense	TGGAATCCTGTGGCATCCATGAAAC
	antisense	TAAAACGCAGCTCAGTAACAGTCC
mmu-miR-181a-5p	sense	AACATTCAACGCTGTCGGTGAGT

### Agilent mouse miRNA microarray

The immature splenic DCs from C57BL/6J mice (6–8 weeks of age) were divided randomly into two groups (0 ng/ml control group and 100 ng/ml group), and were plated into 6-well flat bottom plates at 2 × 10^6^ cell/ml (3 ml per well) in a medium containing 10% FCS at 37 °C in 5% CO_2_ in humidified air. About 4 to 5 hours later, DCs were untreated (0 ng/ml) or were stimulated with HMGB1 (100 ng/ml). Cells were collected after 48 hours. The total RNA was extracted with TRIzol reagent and was quantified with a NanoDrop ND-2000 spectrophotometer (Thermo Scientific), and the RNA integrity was assessed using an Agilent 2100 Bioanalyzer (Agilent Technologies). Sample labeling, microarray hybridization and sample washing were performed according to the manufacturer’s standard protocols. Briefly, total RNA was dephosphorylated, denatured and then labeled with Cyanine-3-CTP. After being purified, the labeled RNA was hybridized onto the microarray. An Agilent Mouse miRNA Microarray, release 21.0, experiment was performed. After being washed, the microarrays were scanned with an Agilent Scanner G2505C (Agilent Technologies), and Feature Extraction software (version 10.7.1.1, Agilent Technologies) was used to analyze the microarray images to obtain raw data. Next, Genespring software (version 11.5; Agilent Technologies) was then utilized to finish the basic analysis of the raw data, which were normalized with the quantile algorithm. The differentially expressed miRNAs were then identified. The threshold used to detect up- and down-regulated genes was an absolute fold change ≥1, The corresponding *P* value was ≤0.05. The target genes of the differentially expressed miRNAs were subsequently predicted by miRANDA software (version v3.3a).

### Reporter plasmid construction and luciferase assays

We cloned the computationally predicted putative cDNA fragment, including the predicted miR-181a-5p binding sites, and its mutant from 441 to 520 nt in the mouse TNF-α-3′UTR (Fig. 3b, the WT sense and the MUT sense) into the MCS (multiple cloning site) of a pmirGLO reporter vector and named the resulting constructs WT and MUT, respectively. As positive controls, miR-181a-5p inhibitors were used. Two duplicates of the miR-181a-5p inhibitor were cotransfected into 293 T cells as a positive control (named PC). Additionally, an empty pmirGLO reporter vector was used as a negative control (named NC). The 80 bp sequence of the TNF-α_3′UTR (Fig. 3b) containing the predicted miR-181a-5p binding sites (Fig. 3a) and its mutant (Fig. 3b) were synthesized by Suzhou Genepharma. The DNA fragments were digested with Sac I and Xho I for 2 hours at 37 °C. The resulting fragments were then subcloned into the Sac I and Xho I sites of a pmirGLO Dual-Luciferase miRNA Target Expression Vector (Promega). 293 T cells were then seeded in 24-well plates at a density of 2.5 × 10^5^ cells per well at 24 hours before transfection. The cells were transfected using Lipofectamine 2000 -according to the manufacturer’s instructions, with FAM-conjugated mimic-NC to observe the transfection efficiency, which should be more than 70% for an effective transfection. Then the 293 T cells were transfected with TNF-α-pmirGLO or mutant of TNF-α-pmirGLO and the mimic of NC or miR-181a-5p. Luciferase assays were performed 48 hours after transfection following the manufacturer’s instructions. The pmirGLO vector is based on Promega dual-luciferase technology, with firefly luciferase acting as the primary reporter for mRNA transcription and Renilla luciferase acting as a control reporter for normalization. Normalized firely luciferase activity (Firefly luciferase activity/Renilla luciferase activity) for each construct was compared to that of the pmirGLO Vector no-insert control. For each transfection luciferase activity was averaged from three replicates.

### miRNA quantitative real-time PCR assays

For quantitative miRNA PCR, total RNA including small RNA was isolated using the TRIzol reagent from mouse splenic CD11c^+^ DCs that were treated with recombinant HMGB1 at different concentrations (0 or 100 ng/ml) and cultured for 48 hours. Reverse transcription and the poly A tail were processed using a miRNA first strand cDNA synthesis (Poly A Tailing) kit (Sangon Biotech), and then, specific primers for miR-181a-5p and the universal PCR primer R (Sangon Biotech) were used to perform real-time PCR (Table 2). All samples were examined in triplicate, and U6 was used to normalize the expression levels of miR-181a-5p by correcting differences in the amount of RNA loaded into qPCR reactions.

### Transfections with miR-181a-5p mimic and inhibitor

The miR-181a-5p mimic, mimic negative control (mimic NC), inhibitor, inhibitor negative control (inhibitor NC), FAM-conjugated mimic-NC, FAM-conjugated inhibitor-NC were obtained by Suzhou GenePharma. (Table 3).

**Table 3:** Sequences used to regulate endogenous miR-181a-5p.

miR-181a-5p mimic	sense	5′-AACAUUCAACGCUGUCGGUGAGU
	antisense	3′-UCACCGACAGCGUUGAAUGUUUU
miR-181a-5p inhibitor	sense	5′-ACUCACCGACAGCGUUGAAUGUU
mimic NC	sense	5′-UUCUCCGAACGUGUCACGUTT
	antisense	3′-ACGUGACACGUUCGGAGAATT
inhibitor NC	sense	5′-CAGUACUUUUGUGUAGUACAA

Splenic DCs from mice were collected and seeded at a cell density of 2 × 10^6^ cells/ml (2 ml per well) in 6-well plates 12 hours before transfection. The cells were transfected using Lipofectamine 2000 according to the manufacturer’s instructions, with FAM-conjugated mimic-NC and FAM-conjugated inhibitor-NC to observe the transfection efficiencies which should be more than 70% for an effective transfection. Then the splenic DCs cells were transfected with mimic, mimic NC, inhibitor and inhibitor NC of miR-181a-5p, respectively. Then the cells were cultured at 37 °C in 5% CO_2_ in humidified air for 48 hours. After extraction of total RNA and reverse transcription into cDNA reverse transcribing, the expression of TNF-α mRNA, which is a target mRNA of miR-181a-5p, was determined with RT-PCR.

### Statistical analyses

Data are represented as the mean ± standard error of the mean (SEM) and analyzed with one-way ANOVA. Unpaired Student’s t-tests were used to evaluate the significance of the differences between groups. P values ≤ 0.05 were considered statistically significant.

## Electronic supplementary material

Supplementary Information

## References

[1] Hotchkiss RS, Karl IE (2003). The pathophysiology and treatment of sepsis. N. Engl. J. Med..

[2] Angus DC, van der Poll T (2013). Severe sepsis and septic shock. N. Engl. J. Med..

[3] Russell JA (2006). Management of sepsis. N. Engl. J. Med..

[4] Banchereau J, Steinman RM (1998). Dendritic cells and the control of immunity. Nature..

[5] Zitvogel L (2002). Dendritic and natural killer cells cooperate in the control/switch of innate immunity. J. Exp. Med..

[6] Miyake Y, Yamasaki S (2012). Sensing necrotic cells. Adv. Exp. Med. Biol..

[7] Thomas JO, Travers AA (2001). HMG1 and 2, and related ‘architectural’ DNA-binding proteins. Trends Biochem. Sci..

[8] Chan JK (2012). Alarmins: awaiting a clinical response. J. Clin. Invest..

[9] Wang H (1999). HMG-1 as a late mediator of endotoxin lethality in mice. Science..

[10] Chen G, Ward MF, Sama AE, Wang H (2004). Extracellular HMGB1 as a proinflammatory cytokine. J. Interferon Cytokine Res..

[11] Dumitriu IE, Baruah P, Bianchi ME, Manfredi AA, Rovere-Querini P (2005). Requirement of HMGB1 and RAGE for the maturation of human plasmacytoid dendritic cells. Eur. J. Immunol..

[12] Yang D (2007). High mobility group box-1 protein induces the migration and activation of human dendritic cells and acts as an alarmin. J. Leukoc. Biol..

[13] Messmer D (2004). High mobility group box protein 1: an endogenous signal for dendritic cell maturation and Th1 polarization. J. Immunol..

[14] Dumitriu IE, Bianchi ME, Bacci M, Manfredi AA, Rovere-Querini P (2007). The secretion of HMGB1 is required for the migration of maturing dendritic cells. J. Leukoc. Biol..

[15] Zhu XM (2009). The effect of high mobility group box-1 protein on splenic dendritic cell maturation in rats. J. Interferon Cytokine Res..

[16] Zhang Y (2011). The potential effect and mechanism of high-mobility group box 1 protein on regulatory T cell-mediated immunosuppression. J. Interferon Cytokine Res..

[17] Chendrimada TP (2005). TRBP recruits the Dicer complex to Ago2 for microRNA processing and gene silencing. Nature..

[18] Chendrimada TP (2007). MicroRNA silencing through RISC recruitment of eIF6. Nature..

[19] Vasudevan S, Tong Y, Steitz JA (2007). Switching from repression to activation: microRNAs can up-regulate translation. Science..

[20] Henke JI (2008). microRNA-122 stimulates translation of hepatitis C virus RNA. EMBO J..

[21] Jopling CL, Schütz S, Sarnow P (2008). Position-dependent function for a tandem microRNA miR-122-binding site located in the hepatitis C virus RNA genome. Cell Host Microbe.

[22] Pedersen I, David M (2008). MicroRNAs in the immune response. Cytokine.

[23] Baltimore D, Boldin MP, O’Connell RM, Rao DS, Taganov KD (2008). MicroRNAs: new regulators of immune cell development and function. Nat. Immunol..

[24] Ansel KM (2013). RNA regulation of the immune system. Immunol. Rev..

[25] Dan C (2015). Modulation of TNF-alpha mRNA stability by human antigen R and miR181s in sepsis-induced immunoparalysis. EMBO Mol Med..

[26] Wu C (2012). microRNA-181a represses ox-LDL-stimulated inflammatory response in dendritic cell by targeting c-Fos. J. Lipid Res..

[27] Tawara I (2011). Interleukin-6 modulates graft-versus-host responses after experimental allogeneic bone marrow transplantation. Clin. Cancer Res..

[28] Lu C (2011). miR-221 and miR-155 regulate human dendritic cell development, apoptosis, and IL-12 production through targeting of p27kip1, KPC1, and SOCS-1. Blood..

[29] Ceppi M (2009). MicroRNA-155 modulates the interleukin-1 signaling pathway in activated human monocyte-derived dendritic cells. Proc. Natl. Acad. Sci. USA.

[30] Sun Y (2011). Targeting of microRNA-142-3p in dendritic cells regulates endotoxin-induced mortality. Blood..

[31] Xing Z (1998). IL-6 is an antiinflammatory cytokine required for controlling local or systemic acute inflammatory responses. J. Clin. Invest..

[32] Liu X (2010). MicroRNA-148/152 impair innate response and antigen presentation of TLR-triggered dendritic cells by targeting CaMKIIalpha. J. Immunol..

[33] Scaffidi P, Misteli T, Bianchi ME (2002). Release of chromatin protein HMGB1 by necrotic cells triggers inflammation. Nature..

[34] Dunand-Sauthier I (2011). Silencing of c-Fos expression by microRNA-155 is critical for dendritic cell maturation and function. Blood..

[35] Sun X, Sit A, Feinberg MW (2014). Role of miR-181 family in regulating vascular inflammation and immunity. Trends Cardiovasc. Med..

[36] Kazenwadel J, Michael MZ, Harvey NL (2010). Prox1 expression is negatively regulated by miR-181 in endothelial cells. Blood.

[37] Remus EW (2013). miR181a protects against angiotensin II-induced osteopontin expression in vascular smooth muscle cells. Atherosclerosis..

[38] Chen CZ, Li L, Lodish HF, Bartel DP (2004). MicroRNAs modulate hematopoietic lineage differentiation. Science..

[39] Li QJ (2007). miR-181a is an intrinsic modulator of T cell sensitivity and selection. Cell..

[40] Neilson JR, Zheng GX, Burge CB, Sharp PA (2007). Dynamic regulation of miRNA expression in ordered stages of cellular development. Genes Dev..

[41] Li G (2012). Decline in miR-181a expression with age impairs T cell receptor sensitivity by increasing DUSP6 activity. Nat. Med..

[42] Henao-Mejia J (2013). The microRNA miR-181 is a critical cellular metabolic rheostat essential for NKT cell ontogenesis and lymphocyte development and homeostasis. Immunity..

[43] Ziętara N (2013). Critical role for miR-181a/b-1 in agonist selection of invariant natural killer T cells. Proc. Natl. Acad. Sci. USA.

[44] Dahlhaus M, Schult C, Lange S, Freund M, Junghanss C (2013). MicroRNA 181a influences the expression of HMGB1 and CD4 in acute leukemias. Anticancer Res..

[45] Kanaan Z (2013). Differential microRNA (miRNA) expression could explain microbial tolerance in a novel chronic peritonitis model. Innate Immun..

