# Supplementary material for: TNF-α mRNA is negatively regulated by microRNA-181a-5p in maturation of dendritic cells induced by high mobility group box-1 protein
Source: Sci Rep. 2017 Sep 25;7:12239. doi: 10.1038/s41598-017-12492-3 (PMC5612954; doi:10.1038/s41598-017-12492-3)
Supplement: Supplementary file 1 — Supplementary Information [file 41598_2017_12492_MOESM1_ESM.pdf]

TNF-α mRNA is negatively regulated by microRNA-181a-5p in maturation of dendritic cells induced by high mobility group box-1 protein

Jing Zhu[1, 2, +], Fu-Li Wang[1, +], Hai-Bin Wang[2, +], Ning Dong[1], Xiao-Mei Zhu[1], Yao Wu[1], Yong-Tao Wang[1, 3], Yong-Ming Yao[1, 4, *]

[1]Trauma Research Center, First Hospital Affiliated to the Chinese PLA General Hospital, Beijing 100048, P.R.China

[2]Department of Clinical Laboratory, First Hospital Affiliated to the Chinese PLA General Hospital, Beijing 100048, P.R.China

[3]Department of Emergency Medicine, Tianjin Medical University General Hospital, Tianjin 300052, P.R.China

[4]State Key Laboratory of Kidney Disease, the Chinese PLA General Hospital, Beijing 100853, P.R.China

*Correspondence and requests for materials should be addressed to Y.-M.Y. (email: c_ff@sina.com)

+These authors contributed equally to this work

Supplementary Information

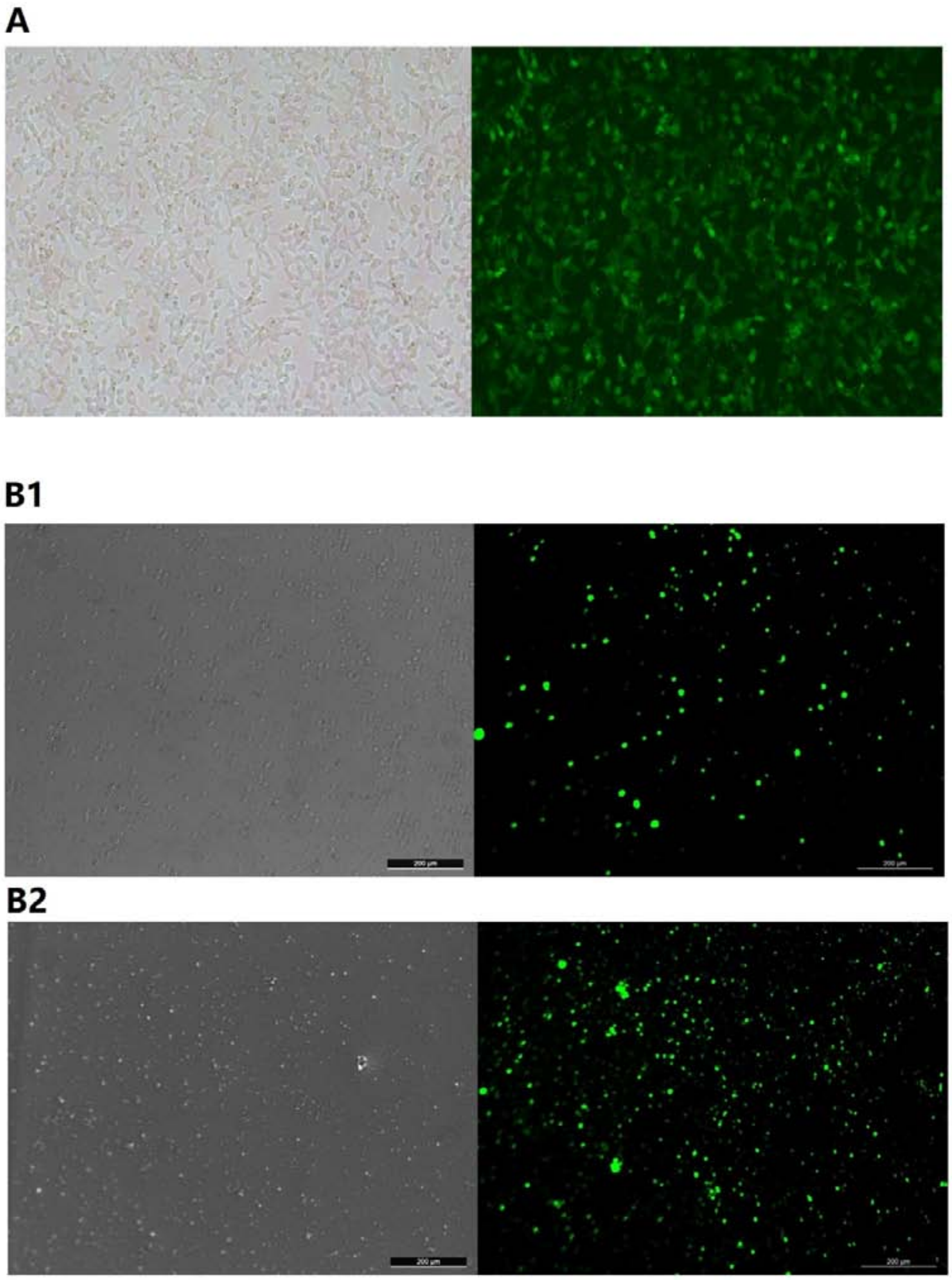

Supplementary Figure S1. The evaluation of transfection efficiency.

A.The 293T cells were transfected with FAM-conjugated mimic-NC. The transfection efficiency was greater than 70%. (The 100 fold view of microscope). **B.** The mice splenic DCs were transfected with FAM-conjugated mimic-NC (shown as B1) and FAM-conjugated inhibitor-NC (shown as B2). Both of the transfection efficiencies were greater than 70%. (The 100 fold view of microscope).

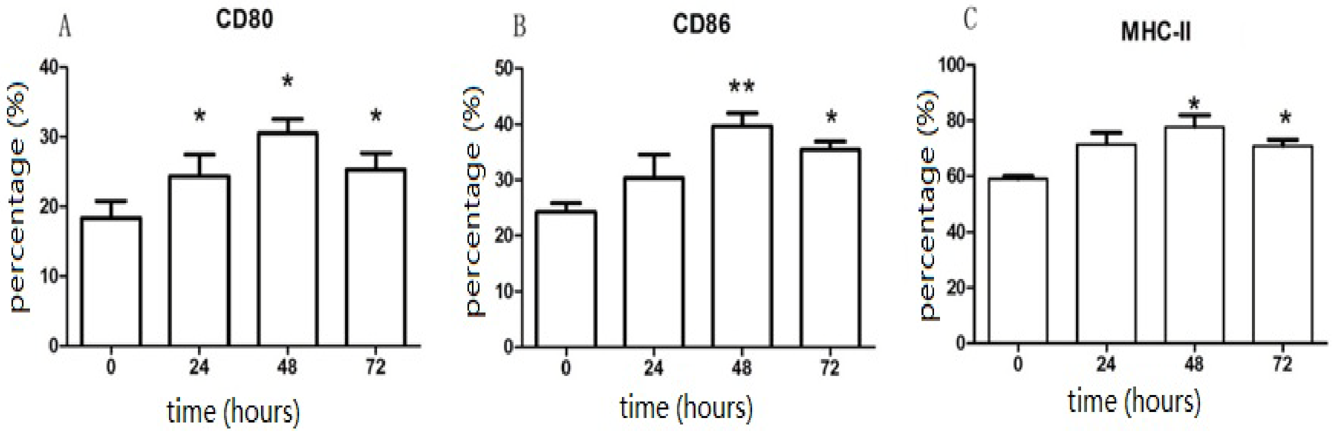

Supplementary Figure S2. Time-course experiments of phenotypic maturation in mouse splenic DCs treated with HMGB1 (100ng/ml). Results of 3 independent experiments were shown as the mean±SEM. *P<0.05 vs. 0 hour group, ** P<0.01 vs. 0 hour group).

The purity and the viability of splenic CD11c^{+}DCs

The purity of splenic CD11c+ DCs was greater than 92% with once MS colume separation, and was greater than 95% with twice MS collume separations. The viability of the eluted cells was greater than 95% by Trypan blue staining.

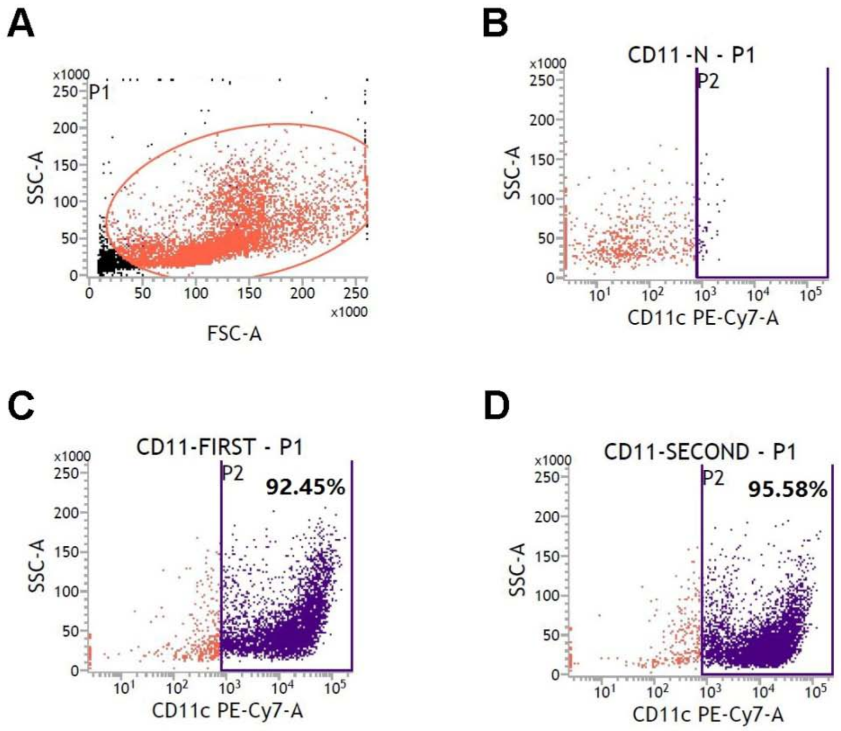

Supplementary Figure S3. Figures of the purity of splenic CD11c+ DCs determined by flow cytometry.

A: The cells in the red cycle were selected to be analyzed. **B**: Negative control without PE-Cy7-CD11c antibody. **C**: The purity of splenic CD11c+ DCs was 92.45% with once MS colume separation. **D**: The purity of splenic CD11c+ DCs was 95.58% with twice MS colume separations.
